# Is Sexual Transmission of Chagas Disease Possible? Evaluating the Evidence and Future Directions

**DOI:** 10.3390/pathogens14111124

**Published:** 2025-11-04

**Authors:** Luis Adrián De Jesús-González, Ignacio Martínez, Bertha Espinoza, Flor Itzel Lira-Hernández

**Affiliations:** 1 Laboratorio de Virología Molecular, Unidad de Investigación Biomédica de Zacatecas, Instituto Mexicano del Seguro Social, Zacatecas 98000, Mexico; flor.lihe@gmail.com; 2 Departamento de Inmunología, Instituto de Investigaciones Biomédicas, Universidad Nacional Autónoma de Mexico (UNAM), Ciudad de Mexico 04510, Mexico; imm@iibiomedicas.unam.mx (I.M.); besgu@iibiomedicas.unam.mx (B.E.)

**Keywords:** Chagas, *Trypanosoma cruzi*, sexual transmission, reproductive system, epidemiology

## Abstract

*Trypanosoma cruzi* is the etiological agent of Chagas disease, traditionally transmitted by triatomine vectors. However, experimental and clinical evidence suggest a possibility that the parasite could also be transmitted sexually. In animal models, *T. cruzi* amastigotes and trypomastigotes have been identified in reproductive tissues and semen, and infection has been experimentally transmitted between mating partners. In humans, the presence of the parasite in menstrual blood, semen, and other genital secretions has been reported, raising the possibility of sexual transmission in both endemic and non-endemic settings. This potential route could contribute to the persistence of infection and the development of congenital cases. Here, we review the current evidence supporting the biological plausibility and experimental demonstration of sexual transmission of *T. cruzi*, and highlight key research priorities to clarify its clinical and epidemiological significance.

## 1. Introduction

Chagas disease was described in 1909 by the Brazilian doctor Carlos Chagas, who identified the causative parasite, the transmitting vector, and the clinical manifestations [1]. The disease was initially considered endemic to America, which is why it was also called American trypanosomiasis. However, human infection has been documented in various European, Asian, and Oceania countries due to endemic population migration to these continents [2,3,4]. In particular, in Mexico, infected people have been found in various states of the Republic, including Mexico City. In Mexico, diagnosis is primarily based on serological screening in blood donors, pregnant women, and the general population, often employing ELISA or Western blot techniques, as recommended by the WHO [5,6]. Epidemiological studies estimate that over one million people in Mexico are infected with *T. cruzi*, though underreporting remains a significant issue [7].

**Figure 1:**
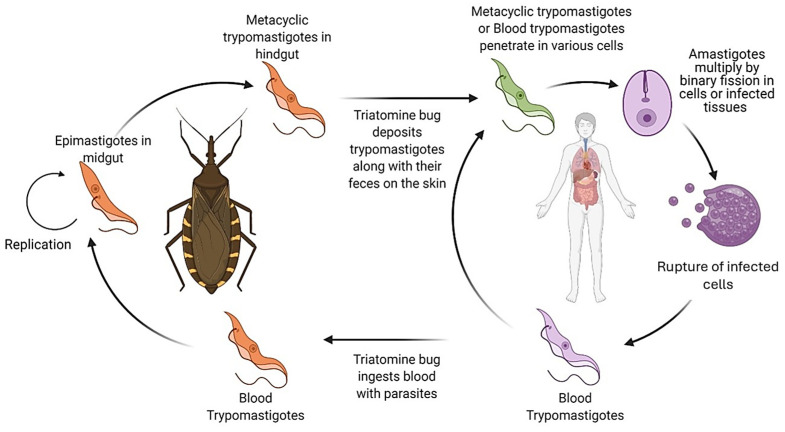
Life cycle in vertebrate hosts (human and non-human mammals). The life cycle of *T. cruzi* involves a vertebrate host and an insect vector (Triatominae).

*Trypanosoma cruzi* Chagas, 1909 (Kinetoplastida, Trypanosomatidae), the etiological agent, is transmitted to humans by hematophagous insects belonging to the family Reduviidae and subfamily Triatominae (in Mexico, there are more than 30 species distributed throughout the national territory). These include *Triatoma dimidiata*, *T. pallidipennis*, *T. barberi*, *T. mazzotti*, and *T. phyllosoma*, among others [8]. These insects, when feeding on the skin, deposit their feces, along with the parasite (Figure 1), which can enter the bloodstream through skin microlesions [7,9,10].

Within the vector, the parasite develops in the midgut as epimastigotes, multiplying through binary fission. Subsequently, it transforms into infectious metacyclic trypomastigotes in the hindgut. These trypomastigotes are deposited along with the insect’s feces on the host’s skin and enter the bloodstream through microabrasions or mucosal membranes. In the vertebrate host, trypomastigotes invade nucleated cells, where they differentiate into intracellular amastigotes. Amastigotes multiply by binary fission and eventually transform into bloodstream trypomastigotes, which cause cell lysis and systemic dissemination. When ingested again by a triatomine during a blood meal, the trypomastigotes restart the cycle within the vector. Moreover, *T. cruzi* infects a wide range of wild and domestic mammals, in which the same intracellular cycle of trypomastigotes and amastigotes occurs across multiple tissues, including reproductive organs. These animal reservoirs play a crucial role in maintaining the parasite in sylvatic and peridomestic cycles, reinforcing the biological plausibility of its presence in mammalian genital tissues [7,9,10].

In the human host, the parasite can infect several types of nucleated cells, which allows it to move throughout the body and establish itself in various tissues. There are two clinical stages associated with the infection: an acute phase that is characterized by nonspecific signs and a chronic stage. Clinical signs, primarily cardiac, can emerge in one-third of patients infected with the protozoan parasite. These manifestations include arrhythmias, conduction system blockages, and heart failure [7,9,10].

The currently available treatment consists of drugs, such as Nifurtimox and Benznidazole, whose use remains debatable since it has low effectiveness and severe side effects. Thus, multiple investigations are underway to identify a more effective compound with a lower risk to patients [11,12,13].

Given the importance of this disease, the International Federation of Associations of People Affected by Chagas Disease, together with the World Health Organization, promoted the establishment of a day dedicated to it. As a result, on 14 April 2020, World Chagas Day was celebrated for the first time [14].

As previously mentioned, the parasite’s main route of transmission is via the vector (via triatomine feces). However, other forms of transmission are recognized, such as transfusion (blood and its derivatives), congenital transmission (from mother to child), oral transmission (which has gained importance in the last decade), and, to a minimal extent, laboratory accidents [15,16,17]. Recently, other possible transmission routes have been considered, such as the participation of other vectors (for example, the bed bug) or the possibility that this parasite may be transmitted during sexual intercourse [18,19,20].

Over the last decades, extensive vector control programs in several Latin American countries have substantially reduced domestic infestation by triatomine insects, decreasing the incidence of vectorial transmission of *T. cruzi* [21]. As a result, non-vectorial routes of infection now account for a growing proportion of new cases. In this context, the possibility of human-to-human sexual transmission becomes more relevant, especially in settings where individuals of reproductive age remain chronically infected despite reduced exposure to vectors.

## 2. Materials and Methods

This narrative review was conducted in accordance with PRISMA recommendations for qualitative synthesis. Scientific literature on *Trypanosoma cruzi* infection related to sexual transmission, reproductive pathology, and congenital outcomes was identified in PubMed, Scopus, and SciELO. Searches included combinations of the terms: “*Trypanosoma cruzi*”, “*sexual transmission*”, “*semen*”, “*placenta*”, “*fertility*”, “*abortion*”, “*pregnancy loss*”, and “*reproductive organs*”, covering publications from 1980 to 2025.

Inclusion criteria comprised original experimental or clinical studies providing evidence of parasite presence, transmission, or pathology in reproductive tissues, gametes, or gestational tissues. Reviews, case reports without parasitological confirmation, and non-peer-reviewed materials were excluded. Reference lists from key papers were also screened to capture additional relevant studies. All included literature was analyzed for methodological quality, parasite detection technique, and reported reproductive or placental outcomes.

## 3. Sexual Transmission of *T. cruzi* in Animal Models

The sexual transmission of various protozoa unrelated to reproductive organs, such as *Entamoeba histolytica* Schaudinn, 1903 (Amoebida, Entamoebidae), *Toxoplasma gondii* Nicolle and Manceaux, 1908 (Eucoccidiorida, Sarcocystidae), and *Trypanosoma brucei* Plimmer and Bradford, 1899 (Kinetoplastida, Trypanosomatidae), has previously been documented experimentally in some animal models [22,23,24].

On the other hand, it has been reported that *T. cruzi* can infect various tissues of the reproductive organs in animal models, both in females (vagina, uterus, and ovary) and in males (foreskin, penis, testicles, epididymis, efferent vessels, seminal vesicle, and prostate) [25]. Amastigotes (intracellular replicative form) and trypomastigotes (infective and circulating form in the blood) of *T. cruzi* have been found in the seminiferous tubules and semen of mice, previously inoculated subcutaneously or intraperitoneally. It is important to emphasize that in chronic infection, the predominant form in tissues is the intracellular amastigote, which is considered less infectious than bloodstream trypomastigotes. Therefore, although sexual transmission has been demonstrated experimentally, the probability under natural chronic conditions is expected to be lower [26,27].

This shows that regardless of the route of entry, some parasite strains can reach the male reproductive organs and semen, opening the possibility of being deposited in the vagina and transmitting the parasite in this way.

This idea was corroborated by intravaginal inoculation of blood trypomastigotes into healthy mice, in which infection with *T. cruzi* and its dissemination to multiple organs were established [28]. Subsequently, it was shown that when infected males mated with healthy females, the latter became infected, as demonstrated by serological tests, detection of T. cruzi genetic material, and the observation of the establishment of amastigote nests in the reproductive organs [18]. It was also observed that males could acquire the infection when infected females mated with healthy males. While not all sexual partners become infected, it has been documented that 20 to 60% of them do [29].

**Figure 2:**
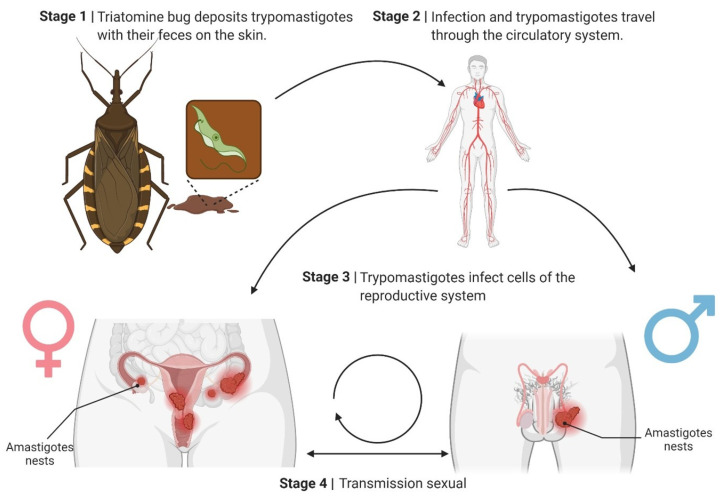
Hypothetical sexual transmission cycle of *Trypanosoma cruzi*. This highlights the potential role of sexual transmission in the epidemiology of Chagas disease.

Viewed carefully, during the acute stage of infection with *T. cruzi*, blood trypomastigotes circulate in the peripheral blood. They could pass from the infected partner to the healthy partner through micro-injuries that occur during sexual intercourse [30]. However, the importance of the work carried out with animal models lies in demonstrating that the infection is transmissible even during the chronic phase of the disease, when the parasite is no longer in circulation but lodged in the reproductive organs as amastigotes [31]. In principle, this implies that the micro-lesions mentioned above can release amastigotes from the infected tissues. These could establish infection in the healthy couple, supported by the infective capacity demonstrated in vitro by this stage of the parasite (Figure 2) [32].

The proposed sexual transmission cycle of *Trypanosoma cruzi* may begin as follows: **Stage 1:** Triatomine bugs deposit *T. cruzi* metacyclic trypomastigotes and their feces on the host’s skin during a blood meal. The parasite enters the host through microabrasions or mucosal membranes. **Stage 2:** The trypomastigotes disseminate through the bloodstream, spreading the infection throughout the body. **Stage 3:** Trypomastigotes invade cells of the reproductive system, including the uterus, ovaries, prostate, and testes. They differentiate into amastigotes and form intracellular nests. **Stage 4:** Sexual transmission occurs when the infected reproductive cells or fluids transfer the parasite to a sexual partner, perpetuating the infection cycle.

This underscores the potential role of sexual transmission in the epidemiology of Chagas disease. A crucial epidemiological implication is that chronically infected individuals may transmit the parasite to their sexual partners during the asymptomatic chronic phase of the disease.

## 4. Sexual Transmission of Chagas Disease in Humans

To date, only one well-documented case has raised strong suspicion of sexual transmission in humans, and although *T. cruzi* DNA and, in some cases, motile parasites have been detected in semen and menstrual blood, these findings alone do not confirm effective transmission. In humans, information regarding the presence of *T. cruzi* in reproductive tissues remains limited, partly because these organs are not the classical targets of Chagas disease pathology and because biopsies or sampling of these tissues entail ethical and medical constraints. Consequently, most available evidence derives from experimental models and indirect clinical observations rather than direct demonstration of transmission events in humans [33,34].

However, the sexual route was proposed by Jörg and Oliva (1980), based on the case of an infected man who lived in the northwestern United States of America, a non-endemic area of the disease and free of triatomines [35]. When analyzing the possible route of infection, it was found that the patient married a young woman who was seropositive for Chagas disease, and that they had sexual intercourse during the girl’s menstruation. Based on this background, Jörg and Oliva (1980) documented *T. cruzi* trypomastigotes’ presence in seropositive women’s menstrual flow, which represents a risk for their sexual partners [35].

Such cases could expose the possibility of a sexual route, in which the menstrual blood of infected women could be the route of infection. The parasites could enter through the urethra of the penis. It has been suggested that the mucous membranes of the penis do not represent a barrier against the parasite due to its thinness, fragility, erosions, and frequent microcracks [25].

The presence of *T. cruzi* trypomastigotes has also been documented in the ejaculate of 18 out of 53 seropositive men (34%), of whom 8 had a positive partner to infection with *T. cruzi* [18]. Likewise, it was demonstrated in murine models that semen from chronically infected male mice containing *T. cruzi* could transmit the parasite to naïve females after vaginal inoculation and to males after intraperitoneal inoculation [18,36].

In addition, the possibility of being infected through oral sex could be raised. It is not unknown that some couples have oral sex even during menstruation, a route of infection mentioned in other parasitosis and viral infections [37,38]. And the risk increases due to the known capacity of the oral transmission of *T. cruzi* [39]. Given this, it is necessary to continue exploring these cases and obtaining more evidence to establish the conditions under which they occur.

## 5. Effects on Fertility, Abortions, and Premature Deaths of Newborns

In addition to the transmission of the parasite from an infected partner to a healthy one, which, by itself, represents a health risk, the possibility that the infection directly affects the reproductive organs should be considered, as reported in animal models of infection with this parasite [40]. It has been documented that the presence of amastigotes in male mice’s reproductive organs during the chronic stage of disease is associated with a decrease in germ epithelium and the release of immature germ cells, thereby favoring infertility [41].

On the other hand, the presence of the parasite in the uterus can affect the gestation process. In animal models, it has been observed that the higher the parasitemia during acute infection in female mice, the less likely it is that an embryo will successfully establish in the uterus [42]. Interestingly, treatment with benznidazole during the acute phase of the infection and before mating drastically reduces the parasitemia and increases embryo implantation and term arrival of the young [43].

Other results have shown that ovulation, fertilization, and first zygote divisions usually occur in infected mice. However, some embryos stop their development during the following divisions, which leads to the formation of abnormal blastocysts that cannot be implanted properly [44]. Likewise, those embryos that manage to implant suffer a delay in their intrauterine growth, and many of them die in the uterus during pregnancy. It has been suggested that this occurs possibly due to a late event of massive ischemic necrosis in the placenta, favored by the presence of amastigotes of *T. cruzi* in this tissue [45,46].

Across both murine and human studies, the severity of reproductive outcomes is closely associated with parasite load or placental invasion. In experimental models, massive placental infection and ischemic necrosis are observed when maternal parasitemia reaches 10^5^–10^6^ parasites/mL, while treatment with benznidazole, which reduces parasitemia by over 90%, restores implantation and fetal viability. In humans, hemoculture positivity (used as a surrogate of high parasitemia) is linked to prematurity, low birth weight, and increased neonatal mortality. However, no quantitative threshold has yet been established to predict placental necrosis or pregnancy loss, underscoring the need for standardized molecular quantification (e.g., qPCR) to define parasite burden and reproductive risk better [47,48,49].

It is important to note that the reproductive outcomes observed in experimental models are influenced by variability in parasite strain, genotype, and host species biology. Different *T. cruzi* genotypes (e.g., TcI, TcII, TcVI) exhibit variation in tissue tropism, parasitemia profiles, and placental invasiveness, leading to distinct effects on implantation, fetal growth, and neonatal survival [50,51]. Similarly, murine strains differ in immune responsiveness and placental architecture, which can modulate susceptibility to infection-induced placental pathology. These factors limit direct extrapolation of experimental findings to humans and may explain the heterogeneous reproductive effects reported in clinical studies [50,52]. Therefore, understanding parasite genetic diversity and host-specific immune and placental traits that shape infection outcomes is essential for accurately interpreting non-vectorial transmission risks, including potential sexual transmission.

A summary of parasite load levels and reproductive outcomes reported in both experimental and human studies is presented in Table 1.

In addition to the above mechanisms, it has also been proposed that the infection of hormone-producing glands may influence the reduction in fertility of the evaluated models, overproduction of inflammatory cytokines in the oviducts or the uterus. More research is needed in this regard and to relate these results to the events that occur in infected humans [44].

For the moment, the only data published with human patients indicate that when evaluating 302 pregnant women with a seropositive test for *T. cruzi* and 302 seronegative women, the former had twice the risk of product loss and a higher incidence of polyhydramnios (excessive presence of amniotic fluid) [56].

In another study with Bolivian women who were seropositive for *T. cruzi*, it was observed that they had almost twice the number of abortions compared to seronegative mothers [54]. Likewise, in mothers who lived in an area where they was exposed to a higher rate of reinfections, parasitemia was higher, and this was related to a higher probability of preterm birth, seropositive products, significantly lower birth weight, respiratory distress syndrome, and higher mortality in the first weeks of life of the product, compared to children born to healthy mothers [53].

Finally, work was carried out in two Mayan communities in the state of Yucatán, Mexico. Women who were seropositive for *T. cruzi* were found to report a higher number of spontaneous abortions. In addition, a higher proportion of children who died at birth and high mortality during the first month of life of the products, compared to seronegative women, were reported [55].

These results could be related to some authors’ proposals that consider Chagas disease as a cause of abortion during the second trimester of pregnancy, which coincides with the increase in parasitemia observed in some pregnant women during this period [47,48,49].

## 6. Risk Factors for Sexual Transmission of *T. cruzi* in Humans

Although vector-borne transmission is the primary route of *T. cruzi* infection, sexual transmission has been hypothesized as a secondary mechanism. Recent studies, however, suggest that this route may be more relevant than previously thought, particularly in contexts where vector exposure is minimal or absent [18,35,36].

### 6.1. Factors That Could Increase Risk

Non-vectorial transmission of *Trypanosoma cruzi* is becoming increasingly relevant as vector control programs reduce triatomine-mediated infections [57]. In this scenario, the potential for sexual transmission deserves attention, particularly in endemic regions and among migrant populations [58]. Global migration and climate change may reshape the epidemiology of Chagas disease, facilitating the parasite’s introduction into non-endemic regions and potentially expanding the range of triatomine species [59]. These environmental and demographic shifts, combined with the persistence of *T. cruzi* in reproductive tissues, underscore the need for vigilance beyond classical transmission routes.

Several biological and behavioral factors may further increase the risk of sexual transmission. High parasitemia during the acute phase, or localized persistence of parasites in reproductive tissues, can increase the likelihood of *T. cruzi* being present in semen, vaginal secretions, or menstrual blood [18,25,26,27,35,36]. Disruption of the genital mucosa through microlesions, inflammation, or coexisting sexually transmitted infections (STIs) may facilitate parasite entry into the partner’s bloodstream [58,60,61]. Unprotected anal intercourse represents an additional risk due to the thin and highly vascularized rectal mucosa [62]. Moreover, coinfections such as HIV, genital herpes, chlamydia, or gonorrhea can exacerbate mucosal inflammation and increase local parasite burden in genital fluids [63,64,65,66]. Sexual practices during menstrual bleeding may also pose a risk, as *T. cruzi* trypomastigotes have been detected in menstrual flow from HIV-positive women [65,66].

Together, these biological, social, and environmental factors justify the consideration of specific risk scenarios, including endemic regions, serodiscordant couples, and sperm or tissue banks lacking *T. cruzi* screening, especially in the context of global mobility and climate-related ecological change.

### 6.2. Public Health Implications

The possibility of sexual transmission of *T. cruzi* poses important public health implications, especially in contexts where traditional routes of transmission have been partially controlled through vector surveillance programs, transfusion control, and perinatal screening. Although evidence for this transmission route is still limited, its mere existence would justify the implementation of preventive strategies in at-risk populations [21].

A critical aspect is the lack of clear regulations regarding *T. cruzi* screening in assisted reproduction settings, particularly in sperm banks [67]. While most donor selection protocols include common pathogens and diseases such as HIV, syphilis, chlamydia, and hepatitis B and C, *T. cruzi* is rarely considered, even in endemic countries [68]. This raises the possibility of inadvertent transmission to recipient women, with clinical implications that may not become apparent until years later.

Another concerning element is the epidemiological invisibility of this transmission route. Since the clinical manifestations of Chagas disease can take years to develop, and many patients remain asymptomatic during the chronic phase, cases resulting from sexual transmission are likely being underdiagnosed or attributed to other sources of infection. This situation limits the response capacity of health systems to detect cases of non-vector-borne transmission [18,69].

Furthermore, the omission of this route in educational campaigns may leave certain groups unprotected, such as serodiscordant couples or young women of reproductive age in endemic regions. Including *T. cruzi* in sexually transmitted infection (STI) screening in selected clinical settings, for example, patients with a history of exposure in endemic areas or with seropositive partners, could improve early detection and reduce secondary transmission [70].

Finally, it is crucial to consider that communication about this potential transmission route also has bioethical implications. Discussing the possibility of sexual transmission without sufficient scientific certainty could unintentionally contribute to stigma, fear, or discrimination toward individuals living with Chagas disease. Therefore, any mention of this route to patients must be approached with caution, emphasizing the current scientific uncertainty and the lack of evidence supporting frequent or effective sexual transmission in humans [71,72].

At this stage, routine sexual-transmission counseling is not warranted, and no public health recommendations should rely on this route as a confirmed mode of transmission. Instead, discussion should be individualized, grounded in informed consent, patient autonomy, and harm-reduction principles, particularly in serodiscordant couples who actively seek guidance. Recognizing the biological plausibility of sexual transmission is relevant for research and clinical awareness, but its role in epidemiology and disease control remains undetermined, and therefore, policy changes should proceed with measured caution.

## 7. Limitations of Current Evidence

Despite growing interest in the possible sexual transmission of *T. cruzi*, the available scientific evidence still presents multiple limitations that make it difficult to draw firm conclusions about its epidemiological relevance in humans. First, most studies supporting this transmission route have been conducted in animal models, primarily mice and hamsters, where infection conditions, parasite dose, and reproductive context do not always reflect human physiological dynamics [25,26,27]. Although these models have shown that the parasite can colonize reproductive organs and be transmitted between partners through intercourse, extrapolation of these findings to human populations requires caution [18].

In humans, the evidence is scarce, fragmented, and, in many cases, anecdotal. Case studies and retrospective analyses suggest transmission between serodiscordant couples without apparent vector exposure, but most of these reports cannot completely rule out other routes of transmission, such as previous transfusions, undetected congenital transmission, or inadvertent oral exposure. Furthermore, many of the available studies have significant methodological limitations, such as small sample sizes, lack of appropriate controls, selection bias, and lack of longitudinal follow-up [18,35,36].

Molecular detection of the parasite in human sexual fluids, while an important advance, does not always correlate with infectivity, as the presence of parasitic DNA does not necessarily imply viability or the ability to establish infection [73,74]. Another limiting factor is the ethical and logistical difficulty of conducting controlled studies on sexual transmission in humans. Designs such as cohorts of serodiscordant couples, most appropriate for evaluating this route, are scarce and complex to implement due to bioethical implications, cultural barriers, and the need for prolonged follow-up [75]. Added to this is the lack of specific funding for research into this non-traditional transmission route, which partly reflects institutional disinterest in exploring phenomena that have not yet been officially recognized in clinical guidelines for Chagas disease control [21,76].

## 8. Conclusions and Future Perspectives

Currently, the available information on the sexual transmission of *T. cruzi* in humans and its impact on fertility remains limited, underscoring the urgent need for studies to explore this route more thoroughly. Despite experimental evidence confirming the presence of *T. cruzi* in reproductive tissues and sexual fluids, as well as its infectivity in animal models, the extent of the risk in humans and its epidemiological significance remain poorly defined. This lack of clarity is further compounded by the absence of systematic surveillance and routine screening in sperm banks and fertility clinics, particularly in endemic regions. As a result, this potential transmission route remains invisible in official statistics, making it impossible to estimate its true burden or its contribution to the overall incidence of Chagas disease.

One pressing concern is that semen donation in private reproductive centers often occurs without standardized regulation, relying instead on confidential agreements between clinics and donors [77]. Remarkably, while donor screening typically includes sexually transmitted infections such as HIV, *Chlamydia trachomatis* (Busacca, 1935) (Chlamydiales, Chlamydiaceae), syphilis, gonorrhea, herpes simplex virus (Herpesvirales, Herpesviridae), and cytomegalovirus, *T. cruzi* is consistently overlooked [18,78,79]. This omission poses a real risk that women undergoing assisted reproduction procedures could receive gametes from infected donors, potentially resulting in long-term clinical consequences. Therefore, a more robust understanding of this transmission pathway is critical to its prevention.

A priority should be the design of prospective cohort studies in serodiscordant couples, in which only one partner is infected, to monitor seroconversion rates under various clinical and behavioral conditions. Such studies should incorporate detailed molecular analyses of genital secretions and blood, enabling the identification of correlations between parasitic load, clinical phase, sexual practices, and effective transmission. Mathematical modeling may also play a key role in predicting transmission scenarios and informing public health interventions in both endemic and non-endemic areas.

On the clinical front, integrating *T. cruzi* testing into the standard screening panels of sexually transmitted infections in reproductive health settings, especially among patients with epidemiological risk factors or reproductive plans, is a necessary step. Active surveillance in reproductive tissue banks and fertility clinics would not only prevent potential cases of inadvertent transmission but also generate valuable epidemiological data on the prevalence of *T. cruzi* in these contexts.

Experimentally, more research is needed to clarify the tissue persistence of the parasite in reproductive organs during chronic infection, as well as the infectivity of the various parasitic forms present in semen or vaginal secretions. The development of biomarkers capable of indicating sexual transmission risk would be a major advance for diagnostics and monitoring. Likewise, validating humanized animal models could be pivotal for testing prophylactic interventions or evaluating the effect of antiparasitic therapies in reducing genital parasite load.

Ultimately, this research agenda must be accompanied by educational campaigns targeting both healthcare professionals and the public. These efforts should aim to raise awareness about this potential transmission route and promote safe sexual practices. Integrating a gender perspective, reproductive rights, and bioethical considerations will be essential to ensure that public health policies derived from these findings are respectful, equitable, and culturally appropriate. Thus, acknowledging and investigating the sexual transmission of Chagas disease will not only expand the scientific understanding of *T. cruzi* but will also strengthen global strategies for the prevention and control of this neglected tropical disease.

Future research should focus on (a) standardizing quantitative methods such as qPCR to assess parasite burden in reproductive and gestational tissues, (b) defining how *T. cruzi* genetic diversity influences tissue tropism and transmission potential, and (c) conducting prospective clinical studies to clarify the epidemiological relevance of sexual transmission in humans.

From a public health perspective, priorities include (a) strengthening screening protocols for *T. cruzi* in sperm and oocyte donation programs and assisted reproduction clinics, (b) providing counseling and testing strategies for serodiscordant couples in endemic and migrant populations, and (c) incorporating non-vectorial transmission routes into Chagas disease awareness campaigns and clinical guidelines.

Together, these measures would support a more comprehensive understanding and mitigation of Chagas disease transmission in the post-vector-control era.

## Figures and Tables

**Table 1:** Parasite load and reproductive outcomes in experimental and human *Trypanosoma cruzi* infection.

Study	Model	Parasite Load (Method/Description)	Observed Outcome	Quantitative Threshold Defined
Mjihdi et al., 2002 [46].	Mouse (BALB/c, acute Tehuantepec strain)	High parasitemia (~10^6^ parasites/mL, acute phase)	Infertility, placental ischemic necrosis, fetal death (80% infertile)	No; necrosis with placental invasion (125× more amastigotes than heart)
Id Boufker et al., 2006 [44].	Mouse (BALB/c, acute infection)	Parasitemia correlated with embryonic arrest (2–8-cell stage)	Pre-implantation failure, abnormal blastocyst outgrowth	No numeric threshold; direct correlation with parasitemia
Solana et al., 2009 [43].	Mouse (benznidazole-treated infection)	Blood/tissue parasite load reduced by 2–3 logs (qPCR)	↓ Placental necrotic foci, ↓ fetal resorptions	No; improvement proportional to >90% reduction
Solana et al., 2002 [42].	Mouse (C3H/HeN; RA vs. K98 subpopulations)	Higher parasitemia in the K98 clone (day 44 p.i.)	↑ Fetal resorptions, no congenital infection	No; higher load linked to resorption
Cencig et al., 2013 [45].	Mouse (TcI, TcII, TcVI)	Acute phase: 10^4^–10^5^ parasites/mL (inocula 10^3^–10^6^)	Intrauterine growth restriction, fetal death; placental necrosis after reinfection	No; TcVI is the most virulent
Torrico et al., 2006 [53].	Human (Bolivia, 724 mother–child pairs)	Positive maternal hemoculture = high parasitemia	↑ Prematurity, respiratory distress, neonatal mortality (OR > 3)	No numeric cut-off; hemoculture used as proxy
Torrico et al., 2004 [54].	Human (Bolivia)	Not quantified; congenital transmission 5–6%	↓ Neonatal mortality (13% → 2%) with reduced population parasitemia	No
Gamboa-León et al., 2014 [55].	Human (Yucatán, Mexico)	Seropositivity 2.3%; no direct load measure	↑ Stillbirth risk (RR 4.7)	No
Hernández-Matheson et al., 1983 [56].	Human (Argentina)	Positive Machado–Guerreiro test (antibodies)	2-fold higher pregnancy loss; ↑ polyhydramnios	No

Note: ↑: increase, ↓: decrease, OR: odds ratio, and RR: risk relative.
